# Adipose Tissue Compartments, Inflammation, and Cardiovascular Risk in the Context of Depression

**DOI:** 10.3389/fpsyt.2022.831358

**Published:** 2022-04-04

**Authors:** Britta Stapel, Maria Jelinic, Grant R. Drummond, Dagmar Hartung, Kai G. Kahl

**Affiliations:** ^1^Department of Psychiatry, Social Psychiatry and Psychotherapy, Hannover Medical School, Hanover, Germany; ^2^Department of Physiology, Anatomy and Microbiology, Centre for Cardiovascular Biology and Disease Research, School of Life Sciences, La Trobe University, Bundoora, VIC, Australia; ^3^Hannover Medical School, Institute for Diagnostic and Interventional Radiology, Hanover, Germany

**Keywords:** adipose tissue, inflammation, major depressive disorder, cardiovascular disease, HPA axis, body composition

## Abstract

The neurobiological and behavioral underpinnings linking mental disorders, in particular, major depressive disorder (MDD), with cardiovascular disorders are a matter of debate. Recent research focuses on visceral (intra-abdominal and epicardial) adipose tissue and inflammation and their impact on the development of cardiometabolic disorders. Intra-abdominal adipose tissue is defined as an endocrine active fat compartment surrounding inner organs and is associated with type 2 diabetes mellitus, a risk factor for the later development of cardiovascular disorders. Epicardial (pericardial) adipose tissue is a fat compartment surrounding the heart with close proximity to the arteries supporting the heart. Visceral adipose tissue (VAT) is an important source of inflammatory mediators that, in concert with other risk factors, plays a leading role in cardiovascular diseases. In conjunction with the behavioral (physical inactivity, sedentary lifestyle), psychological (adherence problems), and hormonal (dysfunction of the hypothalamus–pituitary–adrenal axis with subsequent hypercortisolism) alterations frequently accompanying MDD, an enhanced risk for cardiovascular disorders results.

## Depression and Cardiometabolic Disorders

Major depressive disorder (MDD) is associated with excess mortality (1, 2). This appears to be in part mediated by an increased lifetime risk for obesity-related comorbidities, including cardiovascular disease (CVD), stroke, and type 2 diabetes mellitus (2–4). Overall, the association of cardiometabolic disorders and depression appears bidirectional as has been shown for obesity (5), metabolic syndrome (MetS) (6), type 2 diabetes (7), and CVD (8).

Several contributing factors are proposed to underlie the high prevalence of comorbid severe mental disease (SMD) and CVD, ranging from shared pathological dysregulations of biological systems to adverse health behaviors (9, 10) and limited social and economic support (11–13). Biological pathways that are associated with SMD include pathological alteration in stress response systems (14) as well as systemic chronic inflammation (15), which, in turn, is shown to profoundly impact metabolic pathways that contribute to the observed increase in metabolic conditions that often precede CVD, including obesity, dyslipedemia, insulin resistance, type 2 diabetes mellitus (16, 17). Additionally, adverse health behaviors, including increased tobacco and alcohol consumption, sedentary behavior, and poor diet, are common in people with SMD and contribute to the onset and progression of cardiometabolic disorders (9, 10). Furthermore, people with SMD are less likely to adhere to treatment regimens for conditions associated with increased CVD risk (18, 19), and especially, patients with schizophrenia were reported to be subjected to insufficient physical health care (20). Finally, iatrogenic effects of medications for SMD are described (21, 22).

## Body Fat Distribution and Cardiovascular Disease

Obesity in general is associated with a significant prevalence of cardiovascular risk factors and heightened risk of cardiovascular events (23). However, distinct body fat distributions have previously been shown to profoundly impact cardiometabolic parameters, and accordingly, numerous studies find that alterations in body fat distribution rather than the amount of total body fat increase the risk for CVD (24). In this regard, abdominal obesity, indicated by high waist circumference, is associated with cardiometabolic disease and CVD and constitutes a predictor for mortality even in individuals within the normal weight range (25–29). As increasing evidence indicates abdominal obesity as a cardiovascular risk marker, in addition to BMI (30–32), assessment of waist circumference is frequently recommended in clinical evaluations (33–36).

Of note, the detrimental impact of abdominal obesity on physical health is not limited to cardiometabolic diseases. Albeit less studied compared with its impact on cardiometabolic diseases, abdominal obesity is associated with numerous physical diseases, including an increased risk for several cancer types (37–39), chronic kidney disease (40), dementia, and Alzheimer’s disease (41, 42), and worsened asthma symptomology (43). As these diseases also appear more prevalent in patients suffering from depression, it appears reasonable that pathological increases in abdominal adipose tissue might contribute to this overall elevated risk of various physical disorders in this patient population (44–48).

**FIGURE 1:**
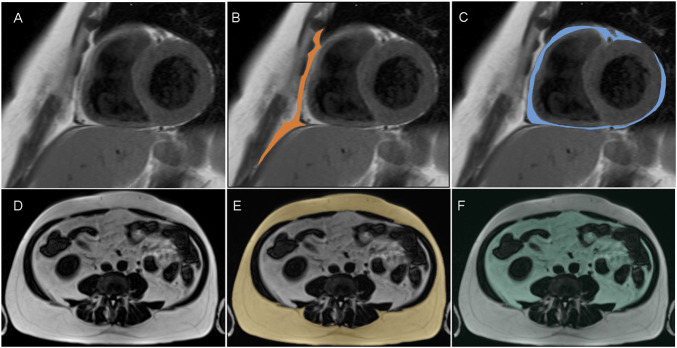
Segmentation of paracardial adipose tissue (PAT; **B**, colored) and epicardial adipose tissue (EAT; **C**, colored) from short axis dark blood t1-weighted turbo spin echo MR images (**A**; representative slice) and segmentation of subcutaneous adipose tissue (SAT; **E**; colored) and visceral adipose tissue (VAT; **F**; colored) from t1-weighted 3-D Volume Interpolated Breathold Examination (VIBE) Dixon MR images (**D**, presentative slice).

Imaging techniques, such as magnetic resonance imaging (MRI) and computed tomography (CT), allow for a more detailed assessment of body composition and a broadened understanding of its role in regard to CVD risk (49, 50). Adipose tissue accumulates predominantly as subcutaneous adipose tissue (SAT) and as ectopic fat deposits that include visceral adipose tissue (VAT) surrounding the inner organs of the abdominal cavity and intrathoracic fat surrounding the heart (51, 52). Based on its location and developmental origin, intrathoracic adipose tissue is classified as epicardial adipose tissue (EAT), located directly on the surface of the heart in close proximity to the coronary vessels; as paracardial adipose tissue (PAT), situated on the surface of the pericardium; or as pericardical adipose tissue (PET) that is quantified as the sum of EAT and PAT (53). Exemplary MR images of SAT, VAT, EAT, and PAT are depicted in Figure 1, and for comparison of MR and CT images regarding the measurements of VAT and SAT, the reader is referred to a dedicated study by Klopfenstein et al. (54). Whereas cohort imaging studies indicate that all fat and ectopic adipose tissue deposits are correlated with each other (55), significant interindividual differences regarding the ratio of SAT to VAT are reported independent of BMI or total adiposity level (56–58). Augmented VAT volumes are shown to exhibit prognostic value in the context of MetS (59–61), and additionally, individuals with excess VAT are found to display high CVD risk independent of BMI (56, 58, 62). With regard to cardiac ectopic fat content, associations of PET with increased BMI, with common risk factors of CVD, and with elevations in lipoprotein particles (63) as well as with a heightened risk of all-cause CVD, atherosclerotic CVD, and heart failure were observed (64). Additionally, correlations of PET with CVD were found after adjustment for age, sex, BMI, and waist circumference, but not when cardiovascular risk factors were included as confounders (65). Similarly, EAT that derives from brown adipose tissue and is demonstrated to secrete cytokines and chemokines (66) is shown to be negatively associated with cardiovascular health scores and to positively correlate with arterial stiffness in patients with type 2 diabetes and CVD (67, 68). Finally, it is demonstrated that EAT thickness is associated with waist circumference, blood pressure, dyslipidemia, and insulin resistance (67, 69), thereby implicating EAT as an early indicator of cardiovascular risk.

## Methods

Based on the recommendations by Bramer et al. (70), we developed the following search algorithm based on the Medical Subject Headings (MeSH) thesaurus by the National Library of Medicine. The search algorithm automatically explores the MeSH terms, that is, searches not only for the terms themselves, but also for all subordinate terms. The following search terms were entered: ((Major depressive disorder [MeSH Terms]) OR (Depression [Title/Abstract]) OR (Depressive disorder [Title/Abstract]) OR (mood disorder [Title/Abstract])) AND ((adipose tissue [MeSH Terms]) OR (body composition [MeSH Terms]) OR (Adipose tissue [Title/Abstract]) OR (Tissue, Adipose [Title/Abstract]) OR (Body Fat [Title/Abstract]) OR (Body Composition* [Title/Abstract]) OR (Composition*, Body [Title/Abstract]) OR (Fat Distribution [Title/Abstract]) OR (Fat Patterning [Title/Abstract]) OR (Abdominal Fat [Title/Abstract]) OR (Abdominal obesity [Title/Abstract]) OR (intra-abdominal fat [Title/Abstract]) OR (intra-abdominal adipose tissue [Title/Abstract]) OR (Epicardial Fat [Title/Abstract]) OR (Epicardial Adipose Tissue [Title/Abstract]) OR (Paracardial Fat [Title/Abstract]) OR (Paracardial Adipose Tissue [Title/Abstract]) OR (Percardial Fat [Title/Abstract]) OR (Pericardial Adipose Tissue [Title/Abstract])).

**FIGURE 2:**
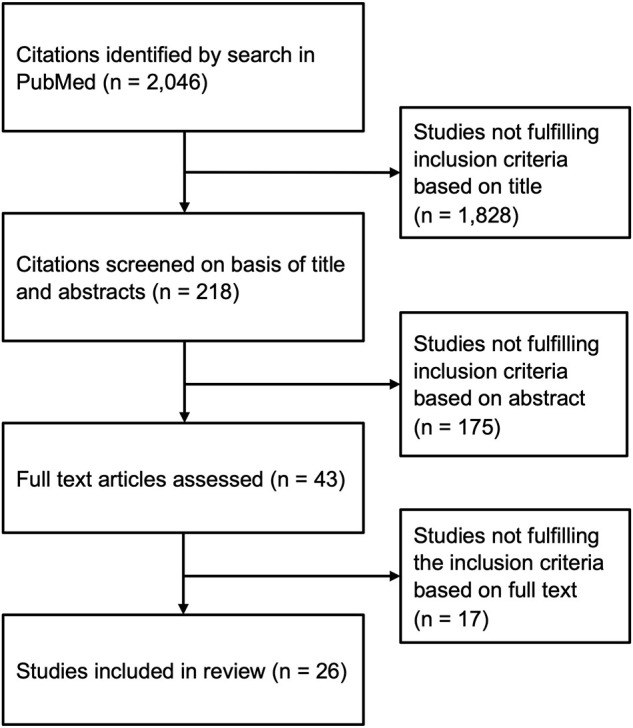
Process of searching for, screening, and selecting eligible articles.

We reviewed articles listed in PubMed/Medline up to December 2021. These included original, review, and systematic review articles and meta-analysis. We additionally scanned the reference lists of identified review articles and meta-analyses for studies fulfilling the inclusion criteria. We identified 25 studies that investigated VAT and/or intrathoracic adipose tissue using CT or MRI measurement in the context of depression. We included one additional study that used echocardiography to detect intrathoracic adipose tissue compartments (94) (Figure 2). All studies included in this narrative review are listed in Table 1.

**TABLE 1:** Summary of MRI and CT studies regarding the association of depression and depressive symptoms with alterations in abdominal tissue distribution.

Study	Sample	Adipose tissue compartment and method of measurement	Finding
Thakore et al. (71)	7 female, NW MDD patients vs. 7 female, NW CTRLs	VAT by CT	Twofold increase in VAT in MDD
Ludescher et al. (72)	10 female MDD patients vs. 12 female CTRLs	VAT by MRI	Significant increase in VAT in MDD; partial correlation of upper abdomen VAT but not of SAT with BDI
Ludescher et al. (73)	11 female MDD patients vs. 45 female CTRLs	VAT and SAT by MRI	Significant increase in SAT and VAT in MDD
Lee et al. (74)	101 premenopausal, OW women	VAT and SAT by CT	Positive association of VAT with Zung’s SDS
Kahl et al. (75)	48 female BPD and/or MDD patients vs. 20 female CTRLs	VAT by MRI	Significant increase in VAT in MDD and BPD/MDD patients
Greggersen et al. (76)	78 premenopausal, female BPD and/or MDD patients vs. 34 CTRLs	VAT by MRI	Significant increase in VAT in BPD/MDD patients
Everson-Rose et al. (77)	409 women (mean age 50.4 years)	VAT and SAT by CT	Significant increase in VAT in presence of a CES-D ≥ 16; positive association of VAT but not SAT with CES-D
Kahl et al. (78)	67 middle-aged MDD patients vs. 26 healthy CTRLs	VAT by MRI	Increase in VAT in male and female MDD patients by trend
Scharnholz et al. (96)	20 MDD patients and 34 CTRLs without HPA axis activation determined by dexamethasone response	VAT by MRI	No significant group differences in VAT
Murabito et al. (95)	1,581 middle-aged women and 1,718 middle-aged men (community-based study)	VAT and SAT by CT	Positive association of VAT but not SAT with CES-D in women only
Alsheri et al. (79)	6,459 middle-aged individuals (mean age 55.4 years) of whom 24.3% had EDS, and 2,745 underwent MRI measurements (community-based study)	VAT by MRI	Graded, positive association of VAT and ESD was substantially diminished following adjustment for total body fat
Cho et al. (80)	4,945 men and 2,293 women, including 333 clinically depressed participants (community-based study)	VAT by CT	Positive association of VAT with BDI in women but not in men
Remigio-Baker et al. (81)	1,017 multiethnic men and women (age 45–87 years), of whom 18.8% had EDS (community-based study)	VAT by CT	Positive association of VAT with EDS in men but not in women, no significant effect of race
Yamamoto et al. (82)	4,333 male employees (community-based study)	VAT and SAT by CT	Positive association of VAT but not SAT with elevated depressive symptoms
Ferrari et al. (83)	101 women with gestational diabetes mellitus	VAT by MRI	Positive association of VAT and BDI-I/II
Xiong et al. (84)	117 postmenopausal, NG, women with depressive symptoms vs. 320 CTRLs	VAT and SAT by MRI	Significant increased VAT and SAT in depressed women; positive association of VAT with SDS
Weber-Hamann et al. (85)	22 postmenopausal MDD patients vs. 23 age-matched female CTRLs	VAT by CT	No significant group differences in VAT
Portugal-Nunes et al. (86)	96 participants (age > 50 years)	VAT and SAT by MRI	Positive association of VAT but not SAT and GDS.
Vogelzangs et al. (87)	2,540 elderly (mean age 73.6 years) non-depressed individuals; baseline and annual follow-up over 5 years	VAT by CT	Positive association of VAT and new onset depressive symptoms in men (GES-D ≥ 10 or AD prescription)
Weber-Hamann et al. (88)	29 elderly MDD patients (mean age 61.5 years) and 14 CTRLs (mean age 61.8 years); baseline and 14-month follow-up	VAT by CT	No significant group differences in VAT at baseline or follow up; significant increase in VAT in MDD but not CTRL over follow-up period
Vogelzangs et al. (89)	2,088 participants (age > 70 years); baseline and 5-year follow-up	VAT by CT	Association of CES-D ≥ 16 at baseline and increased VAT at follow-up
Kahl et al. (90)	27 MDD patients vs. 19 CTRLs	PAT, VAT, and SAT measured by MRI	Significant increase in PAT, increase in VAT by trend in MDD, no group differences in SAT
Kahl et al. (91)	50 MDD patients vs. 25 CTRLs	EAT, PAT, PET, IAT, and SAT by MRI	Significant increase in PAT and PET in males and females with MDD; no group differences in EAT, VAT, and SAT
Kahl et al. (92)	16 chronic and 34 acute MDD patients vs. 25 CTRLs	PAT by MRI	Significant increase in PAT in chronic vs. acute MDD and in acute MDD vs. CTRL; positive association of PAT with BDI and MADRS
Richter et al. (93)	28 female patients with BPD/MDD, 22 with MDD and 26 CTRLs (mean age < 35 years)	EAT by MRI	Significant increase in EAT in BPD/MDD vs. MDD and CTRL. No significant difference in MDD vs. CTRL; no significant association between EAT and MADRS in the combined MDD sample
Kahl et al. (94)	210 young adults (mean age 35.5 years) with ACHD, of which 53 were diagnosed with MDD	EAT by echocardiography	Significant increase in EAT in MDD; positive association of EAT with MADRS

*ACDH, adult congenital heart disease; AD, antidepressant; BDI, Beck’s Depression Inventory; BPD, borderline personality disorder; CES-D, Centre for Epidemiologic Studies Depression, CT, computed tomography; CTRL, control; EDS, elevated depressive symptoms; MADRS, Montgomery-Åsberg Depression Rating Scale; MDD, major depressive disorder; EAT, epicardial adipose tissue; GDS, geriatric depression scale; MRI, magnetic resonance imaging; NG, normoglycemic; PAT, paracardial adipose tissue; PET, pericardial adipose tissue; SAT, subcutaneous adipose tissue; SDS, Self-rating Depression Scale; VAT, visceral adipose tissue.*

## Visceral Adipose Tissue and Depression

A considerable number of studies assessed VAT content using MRI or CT in women with MDD or with regard to depressive symptoms. In this regard, most studies report increased VAT values and a positive association of VAT and depressive symptoms in both pre- as well as post-menopausal subjects. The first study that investigated the association of abdominal fat compartments in the context of depression was conducted by Thakore and colleagues, who found approximately twofold higher VAT content measured by CT in seven normal weight, female MDD patients compared with seven normal weight healthy women (71). Similarly, an MRI study including 10 women with MDD and 12 healthy controls found significantly increased VAT content in the upper abdomen, defined as “a 10 cm slab on the niveau of the upper part the liver” of the MDD group (72). Additionally, partial correlation analysis showed a significant association of VAT in the upper abdomen but not of total VAT or SAT and depressive symptoms assessed by BDI after adjustment for BMI and age (72). A second study by the same group identified increased total body fat, VAT, and SAT levels in 11 female MDD patients compared to 45 healthy women that were matched regarding BMI and waist-to-hip ratio (73). Lee and colleagues recruited 101 overweight, premenopausal women and assessed VAT and SAT by CT. After adjustment for potential confounders, VAT was positively associated with depression scores determined by Zung’s Self-Rating Depression Scale (SDS), whereas no association between SAT and depressive symptoms was found (74). Kahl and colleagues investigated VAT measured by MRI in 48 women diagnosed with MDD and/or borderline personality disorder (BPD) (75). Compared with 20 healthy women, area of VAT was significantly higher in patients comorbid with MDD and BPD and in MDD patients after adjustment for BMI, whereas VAT accumulation was less pronounced in BPD patients without comorbid MDD (75). Similarly, an MRI-based study by Greggersen and colleagues confirms increased VAT content in a sample of 78 premenopausal women with MDD and/or BPD compared with 34 healthy female controls with similar BMI values (76). In a cross-sectional CT study that included 409 middle-aged women, individuals who presented with clinically relevant depressive symptomatology [Center for Epidemiologic Studies Depression (CES-D) score ≥ 16] displayed significantly increased VAT after adjustment for age, race, total percentage of fat, and sex hormone binding globulin (SHBG). Additionally, VAT but not SAT was significantly associated with depressive symptoms after correction for the aforementioned parameters as well as for physical activity and Framingham risk score (77). In a cross-sectional study, Kahl and colleagues assessed muscle mass, adrenal gland volume (AGV), and intra-abdominal adipose tissue in 67 middle-aged, depressed patients and 26 healthy control subjects using MRI. Whereas only male patients, particularly those with chronic depression, displayed reduced muscle mass, both male and female patients showed a trend toward increased VAT content in this study that, however, was not statistically significant (78).

In line with these results, a community-based study that included 1,581 middle-aged women and 1,718 middle-aged men drawn from the Framingham Heart Study (49) found an association between VAT measured by CT and depressive symptoms assessed by CES-D scale in women but not in men after adjustment. Contrarily, no association between SAT and depressive symptoms was observed in either sex (95).

Another community-based study included 6,459 middle-aged individuals of whom 2,475 underwent MRI for assessment of VAT. Using the IDS-SR30 questionnaire, 24.3% of participants with significant depressive symptoms were identified (IDS-SR30 ≥ 14). The study reports a graded, positive association of all measures of abdominal adipose tissue with depressive symptom severity. However, total fat showed a stronger association with depressed mood than other parameters, including waist circumference and VAT, and adjustment for total body fat reduced the magnitude of the association between VAT and depressive symptoms substantially. No significant sex differences were observed in this study (79).

A large community-based study by Cho and colleagues investigated the relationship between VAT assessed by abdominal CT and depressive mood in 4,945 men and 2,293 women. Overall, 333 participants were clinically depressed based on BDI scores. After adjustment for factors of hypertension, diabetes, and BMI, VAT showed a positive association with depressive symptom severity in women but not in men (80).

A study that utilized data from the Multi-Ethnic Study of Atherosclerosis and included 1,017 men and women between the ages of 45 and 87 years analyzed the relation of VAT measured by CT and elevated depressive symptoms defined as a CES-D score ≥16 and/or use of an antidepressant. After adjustment for age and BMI, among others, significantly higher VAT values were detected in men but not in women with elevated depressive symptoms compared with individuals of the same sex and with a CES-D score below 16. Contrarily to sex, no significant effect of race was found (81).

In a study that included 4,333 male employees, VAT and SAT were measured by abdominal CT. Whereas no standard tool was employed to assess depressive symptoms, the utilized questionnaire included items similar to those of Zung’s SDS and the CES-D. After multivariable adjustment, including lifestyle factors, physical activity, and BMI, VAT but not SAT was found to be significantly associated with elevated depressive symptoms (82).

In the context of pregnancy, Ferrari and colleagues assessed VAT by MRI in 101 women with gestational diabetes mellitus and report increased VAT to be associated with an elevated risk for greater depressive symptoms assessed by BDI-I or BDI–II after adjustment for BMI (83).

Regarding the effect of abdominal fat content on depressive symptoms in older adults, an MRI study compared normoglycemic, postmenopausal women with depressive symptoms (*N* = 117) to those without current depressive symptoms (*N* = 320) assessed using SDS. Whereas mean age was comparable in both groups, BMI was higher in the group that was characterized by the elevated depression scores, and similarly significantly higher SAT and VAT values were found (84). Furthermore, logistic regression analysis showed a positive association between depressive symptoms and VAT after correcting for age and life conditions. However, no adjustment for BMI was included (84). Contrarily, in a cross-sectional study that compared 22 postmenopausal women with MDD to 23 age-matched healthy women, no significant group differences regarding VAT measured by CT were found (85). However, effects on VAT content dependent on cortisol status were observed as outlined in the respective paragraph below (85).

A study by Portugal-Nunes et al. included 120 individuals above the age of 50 residing in community dwelling of which 96 underwent MRI measurements for VAT and SAT. After adjustment for main confounders, VAT was found to be a significant predictor of depressive mood determined by the Geriatric Depression Scale (GDS), independent of age. Contrarily, SAT showed no significant association with depression scores. However, age was found to significantly moderate the association of SAT with depressive mood, i.e., higher overall adiposity was positively associated with GDS scores in younger individuals, whereas in older participants, a null or negative association was detected. This was interpreted as a potential protective role of SAT in the geriatric population (86). Similarly, in a sample of 2,540 non-depressed older individuals, Vogelzangs and colleagues found that higher VAT volumes assessed by CT were predictive for new onset of persistent depressive symptoms in men after adjustment for sociodemographic parameters and BMI. Contrarily, the association was not significant in women over a follow-up period of 5 years (87). In a longitudinal CT study, Weber-Hamann et al. assessed VAT content in a sample of 29 elderly patients initially diagnosed with a major depressive episode at baseline and after a 14-month follow-up. VAT content was comparable to that of a respective control sample (*N* = 17) at baseline, and similarly, no significant differences between MDD and the control group were observed at the follow-up time point. Depressive symptoms significantly decreased in the MDD group over the follow-up period, whereas weight gain was comparable in both groups. However, a significant increase in VAT content was only detected in the patient group (88). Finally, a prospective cohort study with a 5-year follow up included 2,088 individuals above the age of 70 years living in a community dwelling. Vogelzangs and colleagues found that depression at baseline, defined as a CES-D score ≥ 16, was associated with a significant increase in VAT at follow-up after adjustment for sociodemographics, lifestyle, diseases, and overall obesity (89).

## Intrathoracic Cardiac Adipose Tissue and Depression

When compared with studies assessing total VAT content in the context of depression, studies investigating intrathoracic adipose tissue compartments are limited. In this regard, a study by Kahl et al. measured intrathoracic PAT volume in addition to VAT content in 27 MDD patients compared with 19 healthy controls (90). After controlling for age, weight, and height, significantly increased PAT values were detected in the MDD group, whereas a trend of VAT volume increase in the MDD group and no effect on SAT was observed (90). A case-controlled study by the same group assessed intrathoracic fat content in 50 male and female inpatients with MDD and 25 healthy controls (91). After adjustment for age, height, weight, and physical activity, increased values for PAT and PET were found in depressed men and women, whereas contrarily, no significant effect of depression was observed with regard to EAT, VAT, or SAT (91). In a further study, Kahl and colleagues assessed PAT volume in a cross-sectional study that included 16 patients with chronic and 34 patients with acute depression compared with 25 healthy controls (92). Male and female patients were included in the study, and PAT volume was assessed by MRI. Overall, PAT volume was increased in patients with chronic MDD compared with patients with acute depression and controls after adjustment for age, height, and weight as potential confounding factors. Additionally, PAT volumes were significantly increased in patients with acute MDD compared with the control group, and similar results regarding PAT content were obtained when results were stratified according to sex. Furthermore, a positive association of PAT with self-reported and clinician-rated depressive scores was observed after adjustment for confounders (92). In a recent study, Richter et al. compared EAT volumes measured by MRI in younger (mean age < 35 years) female patients with MDD and with MDD comorbid with BPD to a healthy control collective of comparable age (93). Contrary to prior studies, EAT volumes were comparable in the MDD and control groups after adjustment for age, BMI, and physical activity. However, patients comorbid with MDD and BPD displayed significantly increased EAT volumes compared with the MDD and control groups. In this study, no significant association between EAT volumes and clinician-rated depression scores was observed in the combined MDD sample (93). Finally, Kahl and colleagues determined ectopic cardiac tissue using echocardiography as well as depressive symptoms in 210 young adults with adult congenital heart disease (ACHD) (94). In this sample, MDD was diagnosed in 53 individuals and was associated with increased EAT values. Additionally, a positive association of EAT and depression severity and BMI and a negative association with physical activity was found (94).

Overall, two recent meta-analyses concluded that MDD as well as self-reported depressive symptoms were associated with enlarged VAT compartments. This association appears independent of sex, age, and method of VAT content assessment (i.e., MRI or CT) as well as method of depression assessment (97, 98). Additionally, the association of VAT with depression remained significant when only studies controlling for BMI were taken into account (98).

## Adipose Tissue Compartments, Hypothalamus–Pituitary–Adrenal Axis, and Depression

Several mechanistic links between obesity and depression, including augmented secretion of cortisol secondary to hypothalamus-pituitary-adrenal (HPA) axis activation, dysregulation of the serotonergic system, alterations in adipokine secretion, and chronic inflammation are proposed (99). The HPA axis as a neuroendocrine system plays a central role in the body’s response to stress. HPA axis hyperactivity in the context of MDD constitutes one of the most common findings in psychiatric research. Assessments of HPA axis activity in the context of depression mostly rely on measurements of cortisol, which constitutes the primary effector of the system and is released by the adrenal glands. A recent systematic review concluded that biologically active cortisol levels, indicated by elevated basal levels for the cortisol awakening response, increased salivary diurnal profiles, urinary and hair cortisol, were elevated in the context of depression, whereas measurement of non-protein-bound cortisol in blood serum resulted in overall inconclusive results (100). The critical impact of the HPA axis with regard to body weight control, obesity, and body fat distribution is highlighted by conditions associated with extreme cortisol levels, i.e., Addison’s disease characterized by hypocortisolism and weight loss and Cushing’s syndrome characterized by hypercortisolism and rapid weight gain (101). Additionally, an association of cortisol and obesity is consistently indicated in the literature. A recent meta-analysis that reviewed studies in humans regarding cortisol awakening response as a state measure for HPA axis activity and hair cortisol as a trait measure found that trait rather than state measures were associated with general and abdominal adiposity in humans (102). Therefore, HPA axis dysfunction might constitute a critical factor that links obesity and depression. In this regard, 9 studies, summarized in Table 2, assessed measures of HPA axis activation in relation to visceral, intrathoracic, or SAT. In these studies, markers for HPA axis activity included (morning) cortisol levels assessed in saliva or blood serum as well as AGV that has been previously identified as a robust proxy marker for chronic cortisol burden in population-based studies (103).

**TABLE 2:** Summary of MRI and CT studies regarding the association of depression and depressive symptoms with alterations in abdominal tissue distribution and changes in parameters of HPA axis activity.

Study	Sample	Parameters of HPA axis activity	Finding
Weber-Hamann et al. (85)	22 postmenopausal MDD patients vs. 23 age-matched female CTRLs	Morning cortisol (saliva) over 7 days	Significant increase in VAT in HC MDD vs. NC MDD patients; no significant difference in VAT between HC MDD vs. NC CTRL
Weber-Hamann et al. (88)	29 elderly MDD patients (mean age 61.5 years) and 14 CTRLs (mean age 61.8 years); baseline and 14-month follow-up	Morning cortisol (saliva) over 7 days	Similar increase in VAT in NC and HC MDD patients over 14-month follow-up; no significant association of VAT and cortisol at baseline or follow-up
Thakore et al. (71)	7 female, NW MDD patients vs. 7 female, NW CTRLs	Morning cortisol	Increased cortisol in MDD vs. CTRL; positive association of VAT and cortisol
Ludescher et al. (72)	10 female MDD patients vs. 12 female CTRLs	AGV by MRI	No significant group differences in AGV; positive association of VAT and AGV in combined sample
Kahl et al. (92)	16 chronic and 34 acute MDD patients vs. 25 CTRLs	AGV by MRI, Morning cortisol (serum)	No significant group differences between chronic and acute MDD and CTRL in AGV; positive association of AGV with PAT and BDI in combined sample; significant increase in cortisol in MDD groups vs. CTRL; positive association of cortisol with BDI and MADRS; no significant association of cortisol with PAT or AGV
Kahl et al. (75)	48 female BPD and/or MDD patients vs. 20 female CTRLs	Morning cortisol (serum)	Significant increase in cortisol in MDD vs. CTRL; no significant association of VAT and cortisol in combined sample
Kahl et al. (91)	50 MDD patients vs. 25 CTRLs	Morning cortisol (serum)	Significant increase in cortisol in MDD vs. CTRL; no significant association of cortisol with VAT, SAT, or intrathoracic fat in combined sample
Kahl et al. (90)	27 MDD patients vs. 19 CTRLs	AGV by MRI, Morning cortisol (serum)	Significant increase in AGV and cortisol in MDD vs. CTRL; positive association of AGV with VAT and PAT; no association of AGV and SAT or cortisol and AGV, SAT, VAT, and PAT in combined sample
Greggersen et al. (76)	78 premenopausal, female BPD and/or MDD patients vs. 34 CTRLs	Morning cortisol (serum)	No significant group differences in cortisol; no significant association of cortisol and VAT
Richter et al. (93)	28 female patients with BPD/MDD, 22 with MDD and 26 CTRLs (mean age < 35 years)	AGV by MRI	Significant increase in AGV in BPD/MDD vs. MDD and CTRL; no group difference between MDD and CTRL in AGV; positive association of AGV with EAT and with MADRS in combined MDD sample
Scharnholz et al. (96)	20 MDD patients and 34 CTRLs without HPA axis activation determined by dexamethasone response	Dexamethasone suppression test, Cortisol awakening response (saliva), 24 h cortisol (urine), AGV by MRI	No significant group differences in HPA axis measures or in VAT

*AGV, adrenal gland volume; BDI, Beck’s Depression Inventory; BPD, borderline personality disorder; CTRL, control; EAT, epicardial adipose tissue; MADRS, Montgomery-Åsberg Depression Rating Scale; MDD, major depressive disorder; MRI, magnetic resonance imaging; HC, hypercortisolemic; NC, normocortisolemic; PAT, paracardial adipose tissue; SAT, subcutaneous adipose tissue; VAT, visceral adipose tissue.*

Weber-Hamann and colleagues assessed cortisol status in 23 postmenopausal inpatients diagnosed with MDD by use of morning saliva cortisol measurements over 7 consecutive days. Patients who presented with hypercortisolism showed an increase in VAT content when compared with depressed patients characterized by cortisol levels within the normal range (85). However, in this study, no significant difference regarding VAT values was observed between hypercortisolemic patients and the normocortisolemic control sample (85). Contrarily, in a longitudinal study by the same group, hypercortisolemic and normocortisolemic depressed patients showed a comparably larger accumulation of visceral fat mass over a time course of 14 months, and no significant association between cortisol measures and VAT was observed either at the baseline measurement or at follow-up (88). Thakore and colleagues found elevated baseline cortisol levels in a sample of 7 female patients with non-psychotic, unipolar major depression, melancholic subtype compared with 7 healthy control individuals. In this study, cortisol levels showed a positive association with VAT content (71).

In a study including 10 women with MDD and 12 controls, AGV was assessed in parallel to VAT content (72). Whereas no significant group difference regarding AGV was observed, VAT correlated significantly with AGV in the combined sample (72).

Similarly, Kahl et al. found no significant differences regarding AGV when comparing patients with chronic or acute depression to healthy controls (92). However, in the combined sample, AGV displayed a positive association with PAT and with self-rated depression scores in a partial correlation analysis controlled for age, height, and weight. Contrarily, whereas morning cortisol levels, assessed in serum samples, were higher in the MDD groups and showed a positive association with self- as well as clinician-rated depressive scores, no association between cortisol levels and PAT or AGV was observed following adjustment for potential confounders (92). This is in line with further studies by Kahl and colleagues that report elevated morning cortisol levels in serum samples from MDD patients compared with the respective control groups (75, 91) but fail to identify significant associations between cortisol levels and VAT content in the combined sample of women with depression and BPD and healthy women (75) and between cortisol levels and VAT, SAT, and intrathoracic cardiac fat content in the context of MDD (91). Contrarily, Kahl et al., report increased AGV in a sample of 27 MDD patients when compared with 19 healthy control subjects after controlling for age, height, and weight (90). In line with the aforementioned studies, a significant positive association of AGV with VAT as well as with PAT was reported for the combined sample with age, weight, and height as covariates, whereas no association between AGV as SAT was observed (90). Additionally, whereas the MDD group displayed increased cortisol levels compared with the control sample, no significant association was observed between cortisol values and parameters of body composition or AGV (90). Contrarily, a study that included female MDD patients with and without comorbid BPD failed to detect significant differences regarding morning cortisol levels in either of the MDD groups when compared with the control sample (76). Additionally, in line with other studies, no significant relation between cortisol levels and VAT was observed in a simple regression analysis (76). Finally, in a recent study, increased AGV was detected in a sample of female patients comorbid with MDD and BPD compared with patients with MDD and an age-matched control group (93). Adrenal gland volume showed a significant association with EAT in the combined MDD sample following adjustment for age, BMI, and physical activity, and additionally, AGV was positively correlated with clinician-rated depression scores (93).

In a dedicated study, Scharnholz and colleagues compared VAT content assessed by MRI in 20 depressed patients who did not display alterations in HPA axis parameters to 34 non-depressed individuals (96). Measures of HPA axis function included dexamethasome suppression test, cortisol awakening response, cortisol excretion, and AGV. All parameters of HPA axis function did not significantly differ between groups following adjustment for age, sex, and BMI. Additionally, no significant effect was observed with regard to VAT volume (96).

Taken together, most studies that assessed parameters of HPA axis function in the context of visceral and intrathoracic adipose tissue measures report cortisol levels and/or AGV with distinct results regarding both measures. Eight studies that report cortisol levels mostly find increased values in the respective patients samples with depression compared with the corresponding control groups (71, 75, 85, 90–92) or included depressed patients characterized by hypercortisolism (88). However, only in one study did cortisol measurements show a significant association with VAT or intrathoracic adipose tissue compartments (71), and one study associated hypercortisolism with increased VAT content (85). Additionally, two studies that assessed morning cortisol levels as well as AGV failed to find a significant association between both parameters (90, 92).

Contrarily, whereas group differences between depressed patients and respective control samples were less homogenous between studies, a positive association between AGV and VAT or intrathoracic fat compartments was reported in four studies (72, 90, 92, 93). The observed differences between the different measures of HPA axis function and their association with parameters of body composition might be in part attributable to the reported shortcomings of single time point measurements of cortisol measurements as a reliable marker of chronic cortisol burden (103). Overall, robust positive associations between VAT and AGV and between measures of intrathoracic adipose tissue and AGV were found in all applicable studies, highlighting the potential impact of chronic HPA axis dysfunction on adverse body composition and subsequent cardiometabolic consequences in the context of MDD.

## Adipose Tissue, Inflammation, and Cardiovascular Risk

Adipose tissue plays an important role in immune regulation. It is well-recognized that adipose tissue is a reservoir for innate and adaptive immune cells (104). In obesity, adipose tissue becomes dysfunctional, promoting inflammation, hyperlipidemia, and insulin resistance, which contributes to the development of type 2 diabetes and CVD (105). A major driver for adipose tissue dysfunction is adipose tissue expansion, a hallmark of obesity (106, 107). In obesity, excess adipose tissue expansion promotes inflammation via a range of mechanisms (i.e., hypoxia, lipolysis, immune cell recruitment, adipokine dysregulation, mechanical stress/pressure, senescence, and adipocyte death) (108, 109). Under conditions of excessive energy intake, adipose tissue expands by both proliferation (hyperplasia) and increased adipocyte size (hypertrophy) (105). In the case of adipocyte hypertrophy, adipose capillary density is reduced. Moreover, hypertrophic adipocytes have a reduced surface area–to-volume ratio, and ultimately, tissue oxygen consumption is diminished, which, in turn, creates a local hypoxic state to propagate ROS production (110). The local hypoxia and subsequent adipocyte death and damage attract macrophages, which initiates a sterile inflammatory response (i.e., inflammation in the absence of infection) (111).

Sterile inflammation is a key contributor to the pathogenesis of CVD, particularly in settings with increased adiposity, such as obesity and type 2 diabetes (112). Sterile inflammation begins when endogenous “danger signals” released from damaged, dead, or dying cells or degrading extracellular matrix (ECM) stimulate pattern recognition receptors on innate immune cells (such as macrophages and dendritic cells) (113). This activates multiprotein oligomers termed inflammasomes, which initiate inflammatory cascades by stimulating the release of cytokines, such as interleukin (IL)-1β and other proinflammatory mediators, including chemokines, predominantly monocyte chemoattractant protein (MCP)-1 in adipose tissue, to recruit additional leukocytes to the site (111, 114). These leukocytes produce more pro-inflammatory mediators, ultimately driving tissue dysfunction and damage. Sterile inflammation is recognized as a key contributor to the pathogenesis of end-organ damage in CVD (115).

Adipose tissue mass and volume can be a powerful tool to predict/measure inflammation. Findings from the Framingham Heart Study indicate that SAT and VAT volumes positively correlate with a number of inflammatory markers (i.e., C-reactive protein (CRP), fibrinogen, IL-6, P-selectin, and tumor necrosis factor receptor (TNFR)-2) (116). In a separate study, VAT but not SAT volume was associated with inflammatory markers [i.e., CRP, MCP-1, and intercellular adhesion molecule (ICAM)-1] in type 2 diabetics (117), and similarly, a correlation of white blood cell count and CRP with VAT but not SAT was found in a cohort of overweight individuals without diabetes, hypertension, or dyslipidemia (118). Indeed, systemic inflammation with increased levels of cytokines appears to be a central feature of metabolically unhealthy obesity, which, in turn, is characterized by an increase in VAT and SAT. Contrarily, metabolically healthy obesity that is characterized by an increase in SAT only does not appear to be associated with an increase in pro-inflammatory markers (105). More recently, Antoniades and colleagues described a CT imaging metric, “the fat attenuation index,” which uses perivascular adipocyte lipid content and size to detect coronary artery inflammation and accurately identify early subclinical coronary artery disease (119).

## Adipose Tissue Compartments, Inflammation, and Depression

Low-grade chronic inflammation is proposed as one of the shared underlying condition in the pathoetiology of depression and CVD (15). Additionally, accumulation of visceral fat constitutes one of the most studied potential mechanisms that links chronic activation of stress systems to inflammatory biomarkers. Several dedicated meta-analyses most commonly identify tumor necrosis factor (TNF)-α and IL-6 as the two cytokines that are significantly upregulated in depressed patients (120–123). Studies based on animal models find TNF-α to interfere with insulin receptor signaling as well as with glucose transport in glucose-sensitive tissues, thereby implicating this cytokine as a key component in obesity-associated insulin resistance (124).

Animal studies also support the link between adiposity (or obesity), inflammation, and depression. Anxiety- and depression-like behavior in obese high fat diet–fed rats is associated with increases in hippocampal proinflammatory cytokine (IL-1β, IL-6, and TNF-α) levels (125). P2X7 receptor antagonist treatment in these obese rats, which blocks inflammasome activation and cytokine production, reverses obesity-induced behavioral abnormalities, indicating that sterile inflammation may be the common link between adiposity and anxiety and depression (126). An additional mechanism that has recently been indicated in the pathoetiology of inflammation-associated depression as well as a potential link between depression and aging processes in the brain on the cellular level is senescence. Cellular senescence is characterized by stable cell cycle arrest and by a pro-inflammatory secretory phenotype (127). This senescence-associated secretory phenotype (SASP), while differing on the basis of cell type, usually is characterized by alterations in the levels of several cytokines and chemokines (127). By measuring this SASP index, Diniz and colleagues found that the interaction of depression and somatic health variables, in particular obesity, was associated with greater cellular senescence in young and middle-aged adults (128). Together with animal studies that link obesity to increased senescence and depression and anxiety-like phenotypes (108, 129), these data suggest senescence as a potential key contributor in the interaction of depression, obesity, and inflammation.

In the setting of human depression, correlations between adipose tissue mass and inflammation have been much less studied, assessment of inflammatory markers was limited to measures of IL-6 and TNF–α and findings to date are somewhat contradictory. We identified 6 studies, summarized in Table 3, that assessed inflammatory cytokines TNF-α and/or IL-6 together with visceral or intrathoracic fat compartments in the context of depression.

**TABLE 3:** Summary of MRI and CT studies regarding the association of depression and depressive symptoms with alterations in abdominal tissue distribution and changes in parameters of inflammation.

Study	Sample	Parameters of inflammation	Finding
Kahl et al. (75)	48 female BPD and/or MDD patients vs. 20 female CTRLs	TNF-α and IL-6 serum levels	Significant increase in TNF-α and IL-6 in MDD groups vs. CTRL; positive association of IL-6 but not TNF-α with VAT in combined sample
Greggersen et al. (76)	78 premenopausal, female BPD and/or MDD patients vs. 34 CTRLs	TNF-α and IL-6 serum levels	Significant increase in TNF-α but not in IL-6 in MDD groups vs. CTRL; positive association of TNF-α but no IL-6 with VAT in combined sample
Kahl et al. (90)	27 MDD patients vs. 19 CTRLs	TNF-α and IL-6 serum levels	Significant increase in TNF-α but not in IL-6 in MDD vs. CTRL; no correlation analyses available
Kahl et al. (91)	50 MDD patients vs. 25 CTRLs	TNF-α and IL-6 serum levels	Significant increase in TNF-α in male but not in female MDD vs. CTRL; no group differences in IL-6 in either sex; no significant association of TNF-α or IL-6 with EAT, PAT, VAT, and SAT
Kahl et al. (92)	16 chronic and 34 acute MDD patients vs. 25 CTRLs	TNF-α and IL-6 serum levels	Significant group differences for TNF-α but not IL-6; no significant association between PAT and TNF-α or IL-6

*BPD, borderline personality disorder; CTRL, control; MDD, major depressive disorder; EAT, epicardial adipose tissue; IL, interleukin; PAT, paracardial adipose tissue; SAT, subcutaneous adipose tissue; TNF, tumor necrosis factor; VAT, visceral adipose tissue.*

For instance, Kahl and colleagues assessed TNF-α and IL-6 levels in conjunction with VAT and/or intrathoracic tissue content in several samples of depressed patients compared with respective corresponding control samples. Increased levels of TNF-α and IL-6 were found in a study that included female patients with MDD with and without comorbid BPD in comparison to healthy controls (75). In this study, IL-6 but not TNF-α serum levels were significantly associated with measures of VAT in a partial correlation analysis corrected for age (75).

Contrarily, Greggersen et al., report increased TNF-α levels in a sample of female MDD patients with and without BPD, whereas IL-6 levels did not differ significantly between groups. Accordingly, a positive association between VAT and TNF-α but not between VAT and IL-6 was observed (76). Similarly, a study that included depressed patients of both sexes found increased serum concentrations of TNF-α in MDD patients, whereas IL-6 levels were comparable to control values. Whereas this study reports a trend toward increased VAT and significantly higher PAT and AGV in the depressed sample, no data regarding the potential association of parameters of body composition and inflammatory cytokines were provided (90).

In a study that assessed parameters of body composition and pro-inflammatory cytokines in depressed patients stratified by sex, increased TNF-α levels were found in male but not in female patients, whereas IL-6 was found comparable to values of the respective control sample in both sexes. This study did not detect a significant association of cytokine levels and any parameter of body composition, including EAT, PAT, VAT, and SAT (91). Finally, Kahl and colleagues assessed PAT volume as well as IL-6 and TNF-α levels in a patient sample subdivided into acute and chronic depression. Group differences regarding TNF-α but not IL-6 were observed, and partial correlation analysis indicated an association between TNF-α and clinician-rated depression scores over the complete sample when controlling for age, weight, and height. However, and in line with most other studies, no significant association was detected between TNF-α and PAT volume (92).

In summary, whereas several studies report increased serum levels of pro-inflammatory cytokines, especially of TNF-α, in their respective MDD samples, only two studies, both including female patients with comorbid BPD, found significant associations of IL-6 and VAT (75) or of TNF-α and VAT (76). Overall, based on the limited number of studies that additionally vary with regard to patient characteristics, assessed adipose tissue compartment and statistical methods used to determine potential correlations, a potential link between increased VAT or intrathoracic adipose tissue and pro-inflammatory cytokines, i.e., TNF-α and IL-6, could not be confirmed.

## Conclusion

**FIGURE 3:**
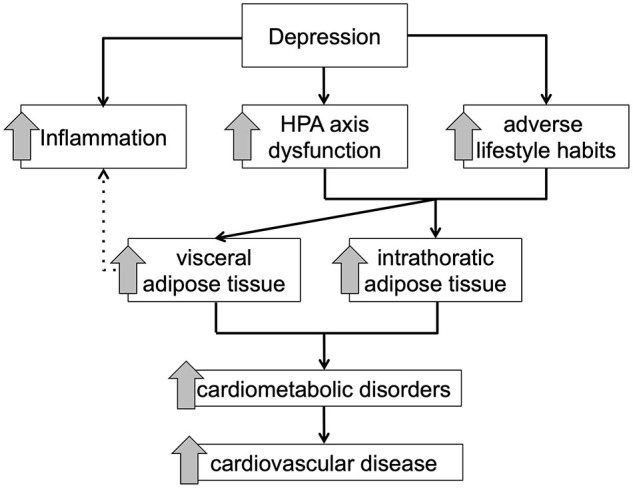
Schematic overview of the proposed mediating effect of adipose tissue compartments between depressive symptomology and cardiometabolic disorder.

MDD as well as self-reported depressive symptoms are associated with severe body composition changes starting in early adulthood, predisposing this group of patients for common physical disorders, such as diabetes mellitus type 2 and cardiovascular disorders. Several MDD-associated mechanisms, such as increased activity of the HPA axis, physical inactivity, poor nourishment, and poor adherence to treatment recommendations, and low-grade inflammation might directly or indirectly worsen this vicious circle, resulting in higher morbidity and mortality rates due to cardiometabolic disorders (Figure 3).

## Author Contributions

BS and MJ: investigation and writing—original draft. GD: writing—review and editing. KK: conceptualization and writing—review and editing. All authors contributed to the article and approved the submitted version.

## Conflict of Interest

KK received speaker honoraria by Janssen, Otsuka, Neuraxpharm, Eli Lilly, Schwabe, Servier, and Trommsdorff/Ferrer; he received an unrestricted grant by Ferrer, ADDISCA. The remaining authors declare that the research was conducted in the absence of any commercial or financial relationships that could be construed as a potential conflict of interest.

## Publisher’s Note

All claims expressed in this article are solely those of the authors and do not necessarily represent those of their affiliated organizations, or those of the publisher, the editors and the reviewers. Any product that may be evaluated in this article, or claim that may be made by its manufacturer, is not guaranteed or endorsed by the publisher.

## Abbreviations

ACHD: Adult congenital heart disease
AGV: Adrenal gland volume
BMI: Body mass index
BPD: Borderline personality disorder
CBT: Congenital behavioral therapy
CES-D: Center for Epidemiologic Studies Depression
CRP: C-reactive protein
CT: Computed tomography
CVD: Cardiovascular disease
EAT: Epicardial adipose tissue
ECM: Extracellular matrix
GDS: Geriatric Depression Scale
HPA: Hypothalamus-pituitary-adrenal
ICAM: Intercellular adhesion molecule
IL: Interleukin
MCP: Chemoattractant protein
MDD: Major depressive disorder
MetS: Metabolic syndrome
MRI: Magnetic resonance imaging
PAT: Paracardial adipose tissue
PET: Pericardial adipose tissue
ROS: Reactive oxygen species
SASP: Senescence-associated secretory phenotype
SAT: Subcutaneous adipose tissue
SDS: Zung’s Self-Rating Depression Scale
SHBG: Sex hormone binding globulin
SMD: Severe mental disease
TNFR: Tumor necrosis factor receptor
VAT: Visceral adipose tissue.
